# Reflections on becoming a senior surgeon

**DOI:** 10.1590/1677-5449.170101

**Published:** 2018

**Authors:** Herbert Dardik

**Affiliations:** 1 Englewood Hospital and Medical Center, General & Vascular Surgery, New Jersey, USA.; 2 Icahn School of Medicine at Mount Sinai, New York, USA.

 Having recently discovered that I have arrived at that point in my life where I am considered a “senior surgeon,” I find it being translated by others as “elderly” or “phasing out” or “becoming a golfer.” As the famous musicologist, Leonard Bernstein, stated on the occasion of his retirement, “I am at the very peak of my decline.” It becomes necessary for those of us arriving at this stage in our lives to make certain critical decisions. Lazar Greenfield, in a magnificent Presidential Address, 1 summarized the major issue confronting not only surgeons but also institutions, the public and all of us as we age and deal with the decline in cognitive function and physical activities. In my opinion, the published manuscript of Dr. Greenfield’s address should be required reading, especially for surgeons at age 60 and beyond. We cannot alter the aging process but we can alter the angle of decline as we shift our activities from the operating room experience to teaching, writing, conferencing and researching. 

 Is there any physician who did not begin his career with that desire, though somewhat naïve, of wanting to be the best and being considered outstanding by his peers? It was sheer excitement to consider adventures in medicine or the challenges of surgery. But of course, as the years roll by, realities set in with the pressure of generating income to cover rent, salaries, premiums and tuitions. At this critical stage, even the role models we aspired to emulate, offered no solace or encouragement but rather justification for activities that should be considered unthinkable and unethical. 

 Each of us needs to take the time and look into ourselves and at ourselves. Are we truly happy with what we are doing? Couldn’t we just revert to some of the goals that we had originally set for ourselves? The answer is an obvious, yes. We need to restore our original orientation and goals. We need to recognize that what we deal with is not simply a commodity but rather human values, life and death, and the quality of existence. Our patients are grateful to us but we must in turn be humbled by their trust and confidence in allowing us to provide care, which at times may be fraught with its own dangers. 

 If I could legislate physician responsibilities, I would include the requirement to be academically productive in a concrete fashion. This not being possible, it behooves each of us to consider such activities on a personal and inspirational basis. Our past performances were monitored and assessed on the basis of written examinations that included preparation of manuscripts. It is equally appropriate that our current clinical performance be assessed in a similar manner. 

 Our organizations and our literature serve as a vehicle for each of us to consider informing our colleagues of achievements, frustrations, and goals. We, the seniors, are responsible for a smooth transition to the next generation as we pass the baton but nonetheless we need to continue planning alternative “races” for ourselves so long as our physical and mental capabilities remain intact. 2 Pablo Casals, in his 90s, was asked why he continued to practice his cello every day. He said, “I am beginning to see improvement.” A clear message to all of us – “keep going,” if you can but be cognizant of the need for change or cessation. The biblical sage, Solomon, noted, “There is an appointed time for everything.” 3

## References

[1] Greenfield LJ (1994). Farewell to surgery. J Vasc Surg.

[2] Rice S (2016). More hospitals screen aging surgeons to make sure their skills are still sharp. Mod Healthc.

[3] The Holy Bible (1995). Ecclesiastes. 3:1.

